# Cyberbullying Among Middle School Students in Sousse, Tunisia: Prevalence and associated factors

**DOI:** 10.1192/j.eurpsy.2025.443

**Published:** 2025-08-26

**Authors:** R. Ayoub, N. E. Ayadi, H. Ben Abid, A. Riahi, K. Faidi, A. Guedria

**Affiliations:** 1 Child and Adolescent Psychiatry Department, Fattouma Bourguiba university Hospital- University of Monastir, Monastir, Tunisia

## Abstract

**Introduction:**

With the increased exposure to social media and the widespread use of technological tools, adolescents are at higher risk of engaging in cyberbullying. Cyberbullying is a specific form of bullying, characterized by persistent aggressive behavior, an imbalance of power, and an intent to cause harm. It occurs through electronic communication, including social media platforms, messaging apps, and other online spaces. In Tunisia, limited data exists on the extent and characteristics of cyberbullying, particularly in the of Sousse.

**Objectives:**

Our study aims to assess the prevalence of cyberbullying among middle school students in the Sousse region and identify the factors associated with this phenomenon.

**Methods:**

This is a cross-sectional, descriptive, and analytical study based on a survey conducted among adolescents attending two middle schools in the governorate of Sousse during the 2020/2021 school year. The participants completed a demographic information sheet, the “Cyberbullying Screening Test,” and the “Resilience Factors Inventory (IFR-40) “

**Results:**

Our population consisted of 238 adolescents, with 63.6% girls and 36.4% boys, giving a sex ratio of 0.57. The average age of participants was between 13 and 15 years old. Our results showed that 38.2% of adolescents used their phones or computers for more than three hours per day, and 32.4% used social media for over three hours daily. The prevalence of cyber victimization was 51.3% within our population. Familial protective factors were present in 80.6% of the sample, individual protective factors in 76.5%, and social protective factors in 68.7%. Our analysis revealed that repeating a grade was statistically associated with cyberbullying (p-value = 0.002). Furthermore, familial protective factors and individual protective factors were significantly correlated with cyberbullying, with p-values of 0.007 and 0.019, respectively. However, no significant association was found with social protective factors (p-value = 0.3).

**Conclusions:**

Our study confirms the concerning extent of cyberbullying among Tunisian adolescents and highlights the need to develop appropriate prevention and intervention strategies to improve youth well-being. Enhancing family and individual resilience appears to be a promising approach for preventing cyberbullying and supporting affected adolescents.

**Disclosure of Interest:**

None Declared

